# Integration of inter-simple sequence repeats with machine learning approach for diversity analysis and authentication of Iranian cotton cultivars

**DOI:** 10.1016/j.bbrep.2025.102435

**Published:** 2026-01-06

**Authors:** Rasmieh Hamid, Zahra Ghorbanzadeh, Bahman Panahi

**Affiliations:** a Department of Plant Breeding, Cotton Research Institute of Iran (CRII), Agricultural Research, Education and Extension Organization (AREEO), Gorgan, Iran; b Department of Systems Biology, Agricultural Biotechnology Research Institute of Iran (ABRII), Agricultural Research, Education and Extension Organization (AREEO), Karaj, Iran; c Department of Genomics, Branch for Northwest & West Region, Agricultural Biotechnology Research Institute of Iran (ABRII), Agricultural Research, Education and Extension Organization (AREEO), Tabriz, 5156915-598, Iran

**Keywords:** Clustering, Combined framework, Cultivar authentication, Decision tree model, Feature selection, Multilocus

## Abstract

Cotton (*Gossypium hirsutum* L.) has experienced extensive breeding in recent decades, leading to a narrowed genetic base that presents challenges for accurate germplasm differentiation and cultivar authentication. This study primarily addresses the lack of reliable, scalable, and interpretable tools for distinguishing closely related Iranian cotton cultivars. To overcome this limitation, the research integrates inter-simple sequence repeat (ISSR) markers with machine learning (ML) algorithms to evaluate genetic diversity and establish diagnostic criteria for cultivar identification. Eighteen commercial cultivars were genotyped using 14 ISSR primers and binary scored data (presence/absence of bands) were used to calculate genetic diversity parameters, including the observed number of alleles (Na), effective number of alleles (Ne), Shannon's information index (I), and expected heterozygosity (He) were calculated. Primers 13, 10, and 26 were identified as the most informative loci, yielding the highest values across diversity parameters. Unweighted Pair Group Method with Arithmetic Mean (UPGMA) clustering and principal coordinates analysis (PCoA) revealed five cultivar groups, with several accessions (e.g., Jahesh, Fakhr, Sahel) showing marked genetic distinctiveness. To enhance cultivar authentication, ISSR data were analyzed using ML classifiers. A decision tree model generated transparent band-based rules, while Random Forest feature selection highlighted key diagnostic loci (Primer24_525, Primer2_766). The combined framework achieved high classification accuracy and reproducibility, enabling reliable discrimination among closely related cultivars. These findings demonstrate the novelty and practical utility of integrating multilocus ISSR markers with ML for cultivar authentication, seed certification, and genetic resource management, while also highlighting previously underexplored genetic diversity that can inform cotton breeding programs in Iran.

## Highlights

• Primers 13, 10, and 26 identified as most informative across diversity metrics.
• UPGMA and PCoA revealed five cultivar groups with distinct genetic profiles.
• Decision tree and Random Forest highlighted key diagnostic loci for classification.
• Combined framework achieved high accuracy and reproducibility in cultivar discrimination.

## 1 Introduction

Cotton (*Gossypium* spp.; 2n = 4x = 52), a member of the Malvaceae family, is **the world's most important natural fiber crop** and also contributes substantially as an oilseed crop. It provides the primary source of natural textile fibers, while cotton seeds yield edible oil and protein-rich byproducts that support food and feed security [1]. Domestication occurred more than 5000 years ago through two independent events: in the Old World (*G. arboreum* and *G. herbaceum*) and the New World (*G. hirsutum* and *G. barbadense*) [2]. ***G. hirsutum* (upland cotton) accounts for the majority of global cotton production due to its adaptability and high yield, whereas *G. barbadense* (Pima or Egyptian cotton) is valued for its extra-long staple fibers** [3]. The Old-World species, found mainly in Asia and Africa, remain **important reservoirs of traits such as pest resistance, drought tolerance, and adaptation to marginal environments** [4]. According to recent reports from the Food and Agriculture Organization (FAO), global cotton lint production exceeds 25 million tons annually, with substantial cultivation concentrated in China, India, the United States, Pakistan, Brazil, and Central Asia. Furthermore, Iran contributes a smaller yet regionally significant role in supporting textile and rural economies (FAO, 2023).

Cotton cultivars and landraces display **wide variation in morphological, physiological, and fiber-related traits** including boll size, lint percentage, fiber length, strength, and maturity [5]. However, the **extensive use of a narrow set of elite cultivars has reduced genetic diversity**, limiting the adaptive potential of modern cotton. The **conservation and molecular characterization of landraces and local cultivars are therefore critical for broadening the genetic base of breeding programs** [[6], [7], [8]]. In Iran, cotton cultivation plays a central role in irrigated agriculture and rural livelihoods, and **current breeding initiatives increasingly focus on evaluating and utilizing indigenous germplasm for sustainable production** [9].

Traditional assessments of genetic diversity based on morphological descriptors are **constrained by environmental influence and limited resolution**. Molecular markers, by contrast, have become indispensable tools for diversity analysis, germplasm characterization, and cultivar authentication [[10], [11], [12]]. A diverse array of marker systems including Random Amplified Polymorphic DNA (RAPD), Amplified Fragment Length Polymorphism (AFLP), Simple Sequence Repeat (SSR), Single Nucleotide Polymorphism (SNP), and Inter-Simple Sequence Repeats (ISSR) has been utilized to quantify allelic richness, assess population structure, and differentiate closely related cultivars. Each system demonstrates varying levels of reproducibility, genome coverage, cost, and technical complexity, making ISSR markers particularly attractive for rapid multilocus profiling in resource-limited breeding programs [13]. ISSRs have been widely applied in genetic diversity studies [14,15], germplasm characterization [16], and genetic mapping [17], providing insights for cotton improvement.

With the increasing scale and complexity of molecular datasets, advanced computational approaches particularly machine learning (ML) have become **powerful tools in plant genetics and breeding**. ML algorithms can uncover hidden patterns, cluster accessions, predict genetic relationships, and classify cultivars from multilocus marker data [18]. **Although SSR and SNP markers are more commonly combined with ML, ISSRs remain highly valuable in resource-limited programs because of their simplicity and reproducibility.** While ML frameworks have been frequently utilized for SSR and SNP datasets, their application with ISSR markers remains limited, particularly in countries where genotyping resources are constrained. Integrating ISSR data with machine learning offers a cost-effective and robust framework for genetic diversity analysis and cultivar authentication. This integration is essential for effective germplasm management, intellectual property protection, and seed certification (Mohammadi et al., 2024, [19]).

Despite the global importance of cotton, there is a significant research gap in integration of ISSR multilocus fingerprints with machine learning classifiers to establish diagnostic rules for differentiating closely related cultivars. This issue is particularly relevant in regions such as Iran, where traditional landraces coexist with modern varieties. The lack of such frameworks hinders accurate diversity assessments and reliable cultivar authentication systems.

This study demonstrates that combining multilocus ISSR marker data with machine learning algorithms can enhance genetic diversity analysis and enable precise authentication of cotton cultivars. The originality of this research lies in creating a transparent, interpretable machine learning-based diagnostic system using cost-effective ISSR markers, providing a practical solution for breeding programs and seed certification in resource-limited settings. The objectives of this study are: (i) to evaluate the genetic variability of Iranian cotton cultivars using ISSR markers, and (ii) to integrate molecular marker data with machine learning approaches to improve diversity analysis and establish reliable systems for cultivar authentication.

## 2 Methods and material

### 2.1 Plant materials

Eighteen commercial cotton (*Gossypium hirsutum* L.) cultivars, **representative of those widely cultivated in Iran**, were used as experimental material. **Seeds were obtained from certified national breeding and research stations.** These cultivars **differ in yield potential, fiber quality, and environmental adaptability, thereby providing a robust basis for diversity assessment and authentication analyses.** A complete list of the cultivars is provided in Table 1.

**Table 1:** Cotton cultivars evaluated in this study, including their names and key agronomic characteristics.

Cultivar	Key Traits/Stress or Agronomic Features	Fiber Quality	Maturity
Golestan	High yield, drought tolerant, VW-resistant	Good fiber length & strength	Medium
Sajedi	Drought tolerant	Good fiber length & strength	Early
Taban	Drought tolerant	Good fiber length & strength	Early
Parto	Drought tolerant	Good fiber length & strength	Early
Sepid	Moderate drought tolerance, medium yield	Good fiber length & strength	Early
Khordad	High yield, moderate drought tolerance	Moderate	Medium-late
Armaghan	Moderate drought tolerance	Moderate	Medium
Kashmar	Moderate drought tolerance	Moderate	Medium
Khorshid	High yield, early maturing, moderate drought tolerance	Moderate	Early
Latif	High yield, yield-stable	Good length & uniformity	Medium
Shayan	Moderate yield, fiber quality tolerant	Good fiber under stress	Medium
Varamin	Drought sensitive, VW-susceptible	Poor fiber under stress	Medium-late
Hekmat	Moderate drought tolerance	Not reported	Medium
Sahel	Drought sensitive, VW susceptible	Poor	Medium-late
Mehr	Drought sensitive, VW-susceptible	Moderate	Medium
Fakhr	High yield, drought tolerant	Good fiber under stress	Early
Bakhtegan	VW susceptible, morphologically characterized	Moderate	Medium
Jahesh	High yield, drought tolerant	Good fiber under stress	Early

### 2.2 DNA extraction

Healthy young leaves were collected from **15-day-old seedlings of each cultivar**. Genomic DNA was extracted using the **cetyltrimethylammonium bromide (CTAB) method** with minor modifications, as described by Ref. [20]. DNA quality was assessed using two complementary methodologies. First, DNA integrity was evaluated through electrophoresis on 0.8 % (w/v) agarose (Agarose, Thermo Fisher Scientific, USA) gels to confirm the presence of high-molecular-weight, intact bands without signs of smearing. Subsequently, DNA purity and concentration were measured spectrophotometrically using a NanoDrop™ 2000 spectrophotometer (Thermo Fisher Scientific, USA) by assessing absorbance at 260 and 280 nm, with samples exhibiting A260/A280 ratios between 1.8 and 2.0 considered acceptable as described in previous studies[8]. The samples were then **diluted to a working concentration of 25 ng/μL and stored at −20 °C for further analysis**.

### 2.3 ISSR-PCR amplification

A set of 14 ISSR primers (synthesized by NovaGene, China) was used for PCR amplification (primer sequences are provided in Supplementary Table S1). The ISSR primers utilized in this study were selected based on their prior validation in cotton and their demonstrated ability to produce clear, reproducible, and polymorphic bands in previous research. Additionally, a preliminary screening was conducted to evaluate amplification efficiency and polymorphism among a subset of cultivars. Only those primers that produced distinct, reproducible, and informative banding patterns were chosen for the comprehensive genotyping analysis.Reactions were carried out in a 25 μL volume containing 25 ng of template DNA, 1 × PCR buffer (Thermo Fisher Scientific, USA), 2.0 mM MgCl_2_ (Thermo Fisher Scientific, USA), 0.2 mM of each dNTP (Thermo Fisher Scientific, USA), 0.5 μM primer, and 1 U of Taq DNA polymerase (Takara Bio Inc., Japan). Amplification was performed in a thermal cycler (Bio-Rad T100™, USA) using the following program: initial denaturation at 94 °C for 5 min; 35 cycles of denaturation at 94 °C for 30 s, annealing at 50–58 °C (depending on the primer) for 45 s, and extension at 72 °C for 90 s; followed by a final extension at 72 °C for 10 min. PCR products were separated on 2 % (w/v) agarose gels, stained with ethidium bromide (Sigma-Aldrich, USA), and visualized under UV illumination using a GelDoc™ XR + system (Bio-Rad, USA) as demonstrated in Ref. [21]**.**

### 2.4 Data scoring and genetic diversity analysis

Clear and reproducible ISSR bands were scored in a binary format, designated as presence (1) or absence (0). A binary matrix was constructed and used for genetic diversity analyses. To evaluate diversity among cotton cultivars, standard population genetic indices were calculated, including the observed number of alleles (Na), effective number of alleles (Ne), Shannon's information index (I), expected heterozygosity (He), and unbiased expected heterozygosity (uHe), and Polymorphic Information Content (PIC) as demonstrated in Ref. [13].

The indices were calculated as follows:

Observed number of alleles (Na):

$$Na=\sum_{i=1}^{k}1\;\left(\text{Nei}\;1973\right)$$

where *k* is the total number of alleles observed per locus.

Effective number of alleles (Ne):

$$Ne=\frac{1}{\sum_{i=1}^{k}P_{i}^{2}}\;\left(\text{Nei}\;1973\right)$$

Where, $P_{i}$ is the frequency of the $i^{th}$ allele.

Shannon's Information Index (I):

$$I=-\sum_{i=1}^{k}P_{i\;}\;\ln\left(P_{i\;}\right)\;\left(\text{Yeung}\;1991\backslash \right)$$

Expected heterozygosity (He):

$$He=1-\sum_{i=1}^{k}P_{i}^{2}\;\left(\text{Nei}\;\text{and}\;\text{Kumar}\;2000\right)$$

Unbiased expected heterozygosity (uHe):

$$uHe=\frac{2N}{2N-1}\times He\;\left(\text{Nei}\;\text{and}\;\text{Kumar}\;2000\right)$$

where N is the sample size (number of individuals).

All calculations were performed in GenAlEx v6.5. Cluster analysis was carried out using the unweighted pair group method with arithmetic mean (UPGMA) in NTSYS-pc v2.1, and principal coordinate analysis (PCoA) was applied to visualize genetic relationships among cultivars as demonstrated in Ref. [11].

### 2.5 Machine learning-based authentication

The binary ISSR marker data were further analyzed using machine learning (ML) approaches to improve resolution in cultivar discrimination and authentication. Feature selection based on Random Forest–derived importance scores was applied to identify the most informative loci contributing to classification. Supervised ML algorithms, including Decision Tree and Random Forest (RF), were implemented in Python v3.9 using the scikit-learn library v1.2 [22,23]. Models were trained on a stratified dataset with tenfold cross-validation, and performance was evaluated using classification accuracy, precision, recall, and F1-score [21,24,25].

## 3 Results

### 3.1 Diversity analysis

The number of different alleles (Na) ranged from 1.375 in Primer 34 to 2.0 in Primers 13 and 10, indicating that these two primers exhibited the highest allelic richness, while Primer 34 amplified the fewest alleles. A similar trend was observed for the number of effective alleles (Ne), which ranged from 1.213 for Primer 32 to 1.879 for Primer 13.

Shannon's information index (I) further supported the enhanced discriminatory power of certain primers. Primer 13 demonstrated the highest information content (0.657), followed by Primers 10, 12, and 26, all of which recorded relatively high values. Conversely, Primer 34 exhibited the lowest Shannon index, reflecting its limited capacity to reveal genetic variation. Expected heterozygosity (He) displayed a comparable trend, with values ranging from 0.143 in Primer 34 to 0.465 in Primer 13. Primers 10, 12, and 26 also yielded high heterozygosity values, whereas Primer 34 consistently showed weak polymorphism.

The unbiased expected heterozygosity (uHe), which accounts for sample size, demonstrated a similar range, from 0.147 in Primer 34 to 0.478 in Primer 13. Polymorphism information content (PIC) values further highlighted differences in primer informativeness. The highest PIC value was recorded for Primer 10 (0.253), followed closely by Primer 13 (0.249) and Primer 32 (0.242). In contrast, Primers 34, 26, and 24 exhibited the lowest PIC values (Table 2).

**Table 2:** Genetic Diversity Indices of Cotton Cultivars Generated Using ISSR Primers. This table presents the observed number of alleles (Na), effective number of alleles (Ne), Shannon's information index (I), expected heterozygosity (He), unbiased expected heterozygosity (uHe), and polymorphic information content (PIC) for each primer, thereby highlighting their relative informativeness and discriminatory power in evaluating genetic variation.

Diversity indices	Primer 24	Primer 34	Primer 2	Primer 4	Primer 26	Primer 12	Primer 13	Primer 32	Primer 10	Primer 19
Na	1.75	1.375	1.666667	1.777778	1.923077	1.9	2	1.5	2	1.8
Ne	1.488587	1.241	1.436148	1.622816	1.628091	1.736707	1.879618	1.213939	1.856237	1.503624
I	0.388724	0.219	0.356014	0.493496	0.519683	0.569884	0.657028	0.238332	0.623665	0.444742
He	0.265293	0.143	0.242802	0.344465	0.353238	0.398726	0.464991	0.149830	0.442567	0.298582
uHe	0.272873	0.147	0.249739	0.354307	0.363330	0.410118	0.478276	0.154111	0.455212	0.307113
PIC	0.2101	0.1901	0.2021	0.1981	0.1967	0.1991	0.2491	0.2424	0.2534	0.2334

## 4 Phylogenetic relationships inferred from ISSR markers

**Fig. 1:**
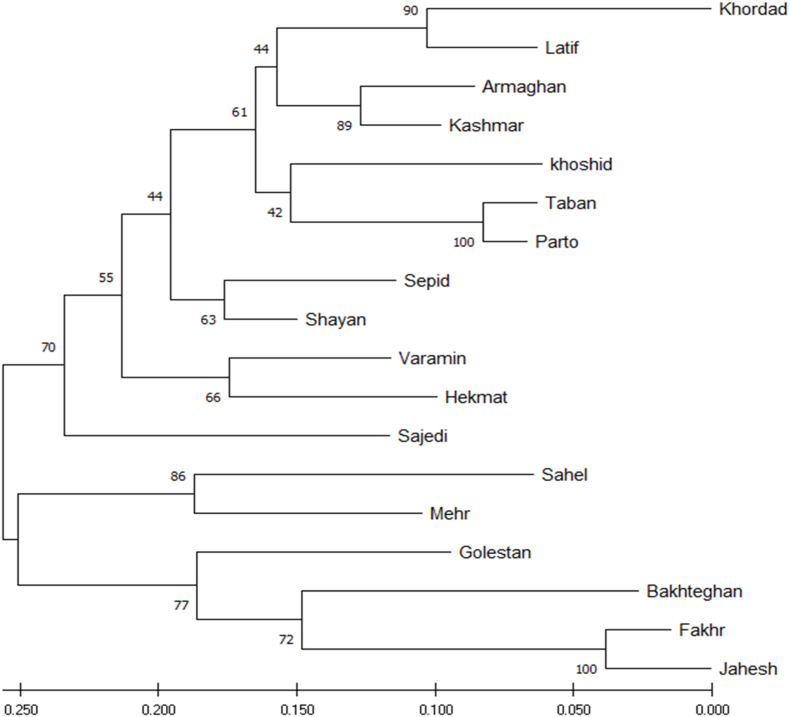
UPGMA dendrogram illustrating the genetic relationships among 18 cotton (*G. hirsutum* L.) cultivars based on ISSR marker profiles. The tree was constructed using Nei's genetic distance, with bootstrap values > 50 % indicated at the corresponding nodes.

The ISSR-based UPGMA dendrogram grouped the 18 Iranian cotton cultivars into **five distinct clusters**, with varying levels of bootstrap support and genetic divergence (Fig. 1). Khordad and Latif formed a strongly supported terminal pair (bootstrap ≈ 90), joined by Armaghan and Kashmar (bootstrap ≈ 89), indicating a genetically cohesive subgroup. Khoshid clustered with the Taban–Parto pair (**bootstrap = 100**), reflecting close kinship. **Sepid and Shayan grouped together (bootstrap ≈ 63) and were subsequently associated with Varamin, Hekmat, and Sajedi, representing an intermediate cluster within the dendrogram.** Sahel, Mehr, and Golestan formed another coherent group, with the **Sahel–Mehr pair strongly** supported **(bootstrap ≈ 86).**

ISSR markers provided sufficient resolution to delineate biologically meaningful clusters. Terminal pairs consistently showed high bootstrap support, reflecting recent common ancestry or shared breeding histories, whereas deeper internal nodes displayed moderate to low support (42–66), warranting cautious interpretation.

**Fig. 2:**
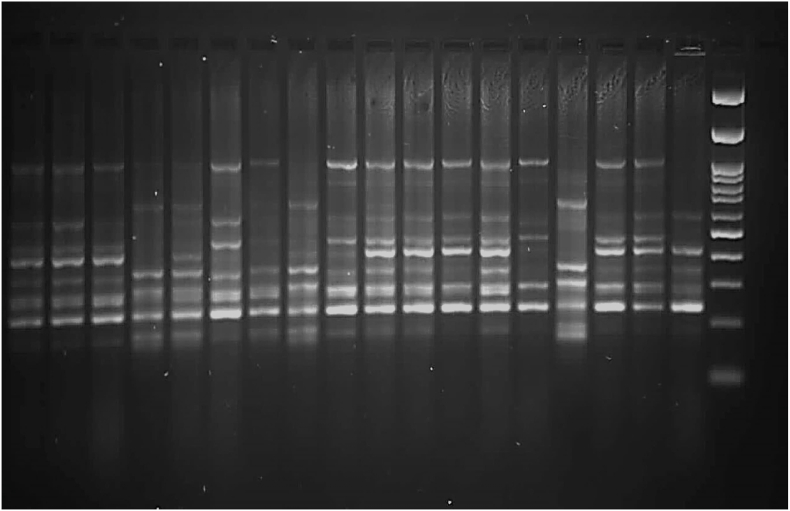
ISSR amplification profiles generated by selected primers across 18 Iranian cotton cultivars, resolved on a 2 % agarose gel. Lanes correspond to individual cultivars as listed in Table 1. M = 100 bp DNA ladder (molecular size marker). Distinct polymorphic bands were scored (1 = presence, 0 = absence) to construct the binary data matrix used in diversity and clustering analyses.

The pattern of low genetic distances within terminal pairs, coupled with moderate divergence among major clusters. Representative ISSR amplification patterns are shown in Fig. 2, showing clear polymorphic banding across cultivars that formed the basis for the diversity matrix.

### 4.1 Principal coordinates analysis

**Fig. 3:**
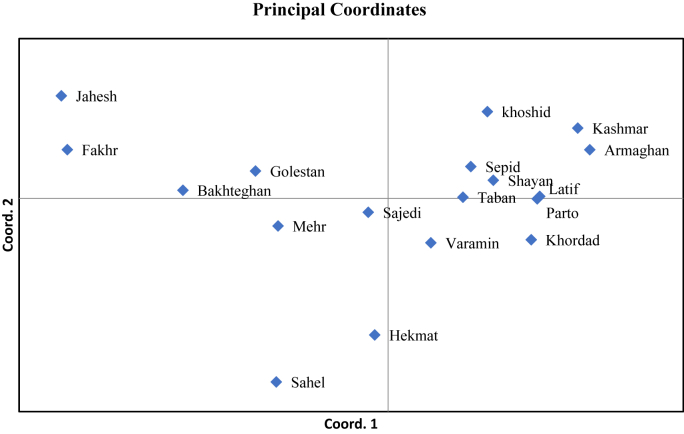
Principal coordinates analysis (PCoA) plot of 18 cotton (*G. hirsutum* L.) cultivars based on ISSR marker data. Coordinate 1 and Coordinate 2 explain 21.6 % and 14.3 % of the variation, respectively (35.9 % cumulative). Cultivars are color-coded according to their UPGMA cluster to facilitate comparative interpretation.

Principal coordinates analysis (PCoA) effectively illustrated the genetic relationships among the 18 Iranian cotton cultivars. The first two coordinates accounted for 21.6 % and 14.3 % of the total genetic variation, respectively, resulting in a cumulative explanation of 35.9 %. The scatterplot revealed a prominent cluster comprising Kashmar, Armaghan, Latif, Sepid, Shayan, Parto, Taban, and Khordad, which were closely positioned within the positive region of Coordinate 1, indicating a high degree of genetic similarity. In contrast, Jahesh and Fakhr were distinctly separated along the negative axis of Coordinate 2, highlighting their genetic divergence from the main cluster. Sahel and Hekmat also occupied isolated positions along the negative ends of Coordinates 1 and 2, respectively (Fig. 3). Golestan, Bakhteghan, Mehr, Sajedi, and Varamin exhibited intermediate placements.

### 4.2 Decision Tree–Based authentication of cotton cultivars using ISSR marker

The integration of ISSR markers with a **machine learning–driven decision tree algorithm provided an effective framework for cultivar authentication.** While traditional approaches such as dendrograms or principal coordinates analysis (PCoA) offer valuable insights into genetic diversity, they often lack the resolution for **unambiguous cultivar assignment. In contrast, decision trees translate ISSR banding patterns into hierarchical rules, allowing explicit and reproducible classification of genotypes.**

**Fig. 4:**
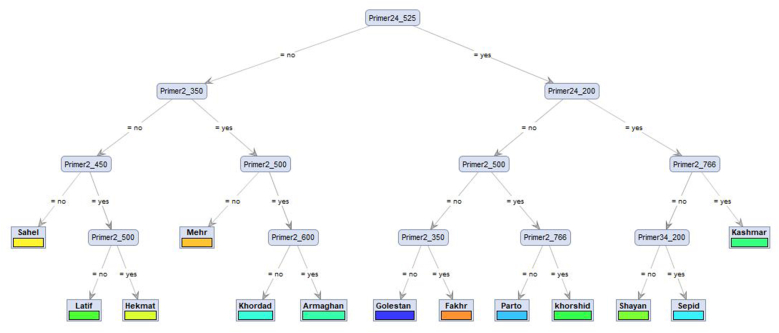
Decision tree illustrating the classification of 18 cotton (*G. hirsutum* L.) cultivars based on ISSR marker profiles. The branching structure demonstrates how specific primer–band combinations (e.g., Primer24_525, Primer2_766) contribute to genetic differentiation and cultivar authentication.

The root node, defined by **Primer24_525**, emerged as the most informative diagnostic locus. Subsequent loci, including **Primer2_350, Primer24_200, Primer2_766, and Primer34_200**, progressively refined cultivar assignment. **For example, the left branch, characterized by the absence of Primer24_525 and partitioned by Primer2_350, grouped cultivars such as Latif, Hekmat, Mehr, Khordad, Armaghan, and Sahel. Conversely, the right branch, defined by the presence of Primer24_525 and further split by Primer2_766, identified Kashmar, Sepid, Shayan, Parto, Khoshid, and Golestan (**Fig. 4**).** This framework demonstrates that combining ISSR markers with decision tree algorithms yields a transparent, rule-based authentication system that avoids the arbitrary thresholds inherent to clustering methods. The explicit marker–band rules ensure reproducibility and facilitate practical application in breeding programs, seed certification, and germplasm management. Importantly, diagnostic loci such as Primer24_525 and Primer2_766 can be developed as molecular signatures for cultivar authentication and genetic resource protection.

The classification performance of the model across 18 cotton cultivars is presented in Table 3. Several cultivars, including *Kashmar*, *Armaghan*, *Taban*, *Khordad*, *Jahesh*, *Fakhr*, *Golestan*, *Bakhteghan*, *Mehr*, *Sajedi*, *Varamin*, and *Khoshid*, achieved perfect accuracy (precision = 1.00, recall = 1.00, F1-score = 1.00), indicating that all instances were accurately classified without any false positives or false negatives. In contrast, cultivars *Sepid*, *Shayan*, and *Hekmat* exhibited moderate performance, attaining a recall of 1.00 but a lower precision of 0.50, resulting in F1-scores of 0.67. This pattern indicates that while the model accurately identified all true instances of these cultivars, it also misclassified some instances from other classes as belonging to them. Notably, cultivars *Latif*, *Parto*, and *Sahel* were completely misclassified, as evidenced by zero values for precision, recall, and F1-score, signifying that none of their instances were correctly predicted. Overall, the model achieved an accuracy of 83.3 % (15/18), demonstrating a strong classification capability for the majority of cultivars, although performance was limited for a small subset, potentially due to overlapping features or insufficient distinguishing information.

**Table 3:** Performance metrics for the classification model applied to ISSR data, showing precision, recall, F1-score, and accuracy for each class, which summarize the model's prediction quality across all cultivars.

Cultivar	Precision	Recall	F1-score
Kashmar	1.00	1.00	1.00
Armaghan	1.00	1.00	1.00
Latif	0.00	0.00	0.00
Sepid	0.50	1.00	0.67
Shayan	0.50	1.00	0.67
Parto	0.00	0.00	0.00
Taban	1.00	1.00	1.00
Khordad	1.00	1.00	1.00
Jahesh	1.00	1.00	1.00
Fakhr	1.00	1.00	1.00
Sahel	0.00	0.00	0.00
Hekmat	0.50	1.00	0.67
Golestan	1.00	1.00	1.00
Bakhteghan	1.00	1.00	1.00
Mehr	1.00	1.00	1.00
Sajedi	1.00	1.00	1.00
Varamin	1.00	1.00	1.00
Khoshid	1.00	1.00	1.00

**Overall accuracy** = (Number of correct predictions/Total predictions) = 15/18 ≈ **83.3 %**.

## 5 Discussion

Cotton possesses a narrow genetic base due to intensive breeding practices and the prevalent use of a limited number of elite cultivars. This situation underscores the necessity of molecular characterization of genetic diversity for effective breeding and conservation efforts [23,26]. ISSR markers are particularly valuable for such analyses, as they are cost-effective, reproducible, and capable of revealing multilocus polymorphisms even among closely related cultivars [15]. This study employed ten ISSR primers to assess genetic diversity among 18 Iranian cotton cultivars, uncovering significant variation in their ability to detect polymorphism.

A key finding of this study was the broad range of diversity index values among the primers, reflecting variations in their discriminatory capacity. Primers 13 and 10 demonstrated the highest values for Na (2.0), Ne (up to 1.879), Shannon's index (0.657), and He (0.465), indicating that these primers amplified a greater number of alleles with a more equitable distribution across cultivars. In contrast, Primer 34 exhibited the lowest values across most indices (e.g., Na = 1.375, He = 0.143), confirming its limited informativeness. The comparison between Primers 13/10 and Primer 34 illustrates how allelic richness (Na) interacts with allele frequency balance (Ne and He) to determine overall marker informativeness. Higher Na signifies greater allelic variation, while elevated Ne and He reflect a more uniform allele distribution; collectively, these factors lead to increased Shannon's index and PIC values. Consequently, the primers with the highest diversity indices were also the most effective in discriminating among cultivars.

These results align with previous findings in cotton and other crops, where specific ISSR primers yield more polymorphic profiles and balanced allele frequencies [[27], [28], [29]]. The notable efficacy of Primers 13, 10, and 26 in our study indicates that these markers effectively capture both allelic richness and evenness, thereby enhancing distance-based clustering and improving the reliability of genetic relationship assessments.

The UPGMA dendrogram classified the cultivars into five primary clusters. Terminal groupings, such as Khordad–Latif and Taban–Parto, were strongly supported by high bootstrap values, indicating recent shared breeding origins. Several cultivars, including Sahel, Jahesh, and Fakhr, highlight genetic divergence within the Iranian germplasm. These cultivars come from different geographic regions and possess unique breeding histories. For example, Sahel is derived from southern Iranian breeding programs adapted to arid conditions, while Jahesh and Fakhr were developed in northern and central regions with different agroclimatic conditions. This separation accounts for their low similarity values in the dendrogram and distinct positioning in the PCoA plot.

The Principal Component Analysis (PCA) elucidated the relationships among cultivars, accounting for 35.9 % of the total variation through its first two axes. Cultivars exhibiting moderate diversity values, such as Primers 24, 12, and 26, formed a central cluster, while those with extreme values, particularly Jahesh and Fakhr, were distinctly separated. This pattern indicates that cultivars contributing significantly to the diversity index, as evidenced by Primer 13 and Primer 10, play a crucial role in driving differentiation in multivariate space. Conversely, cultivars associated with low-diversity primers clustered closely together, reflecting limited genetic variability. Therefore, the PCA not only corroborates the results obtained from the UPGMA analysis but also illustrates how marker-based diversity indices manifest in spatial genetic separation.

A significant contribution of this study is the implementation of a decision tree model for cultivar authentication. While traditional distance-based methodologies summarize relationships, they do not provide explicit classification rules. The decision tree successfully identified critical diagnostic loci, such as Primer24_525 and Primer2_766, which consistently distinguished major cultivar groups. These loci correspond to primers with higher diversity indices, highlighting that the most polymorphic markers enhance diversity resolution and serve as effective predictors in machine learning-based authentication. This correlation between diversity indices and model performance underscores the biological significance of highly polymorphic loci.

Earlier studies have emphasized the utility of machine learning in seed purity testing and cultivar identification [30]. Our findings extend this evidence to ISSR markers, demonstrating that even dominant markers can achieve high authentication accuracy when prioritizing the most informative loci, characterized by the highest Na, Ne, He, and PIC values. This emphasizes the importance of selecting high-performing ISSR primers in the development of molecular identification systems.

Despite the strengths of this approach, ISSR markers possess inherent limitations. As dominant markers, they cannot distinguish between heterozygous and homozygous states, potentially underestimating genetic diversity [30,31]. Furthermore, their inability to detect fine-scale variation may cause cultivars separated by low genetic distances in our dendrogram to appear more similar than they truly are [32,33]. Future integration of high-resolution markers such as SSRs, SNP arrays, or GBS datasets would enhance the patterns observed and clarify relationships among closely related genotypes.

Agreement between the decision tree and distance-based analyses enhances confidence in both the diagnostic loci and the inferred genetic relationships. Discrepancies, on the other hand, highlight cases where a limited number of high-impact loci disproportionately influence classification. This complementarity underscores the value of integrating machine learning with conventional population genetic analyses, as each method provides unique insights into cultivar structure and marker informativeness. Importantly, while SSR and SNP markers are generally considered more robust for ML applications due to their co-dominant nature, our study shows that ISSR markers remain highly relevant in resource-limited programs, where their simplicity, reproducibility, and cost-effectiveness are significant advantages [34].

From an applied perspective, these findings have direct implications for cotton breeding and germplasm management. The identification of highly informative ISSR primers (e.g., 13, 10, and 26) enables the development of diagnostic marker panels for cultivar authentication, consistent with recent reports demonstrating the effectiveness of ISSRs in differentiating cotton varieties [35]. When combined with machine learning classifiers, such panels could be applied in seed certification systems to ensure varietal purity and minimize genetic admixture in commercial seed lots, in line with recent advances in cultivar identification using spectral and ML approaches [36]. Furthermore, the recognition of genetically distinct cultivars, such as Jahesh and Fakhr, provides valuable sources of diversity for broadening the genetic base of breeding programs, supporting the development of improved cultivars with enhanced yield potential, fiber quality, and resilience to biotic and abiotic stresses [37]. The integration of ISSRs with ML also represents a cost-effective and operationally feasible framework for germplasm conservation and resource management in cotton, while complementing efforts to establish standardized DNA fingerprint databases using high-resolution markers such as SSRs and SNPs [7,[38], [39], [40], [41], [42]].

## 6 Conclusion

In this study, we assessed the genetic diversity of 18 Iranian cotton cultivars using ten ISSR primers and integrated these findings with machine learning-based decision tree analysis for cultivar authentication. The highest diversity values were observed for Primers 13, 10, and 26, indicating their superior ability to capture both allelic richness and balanced allele frequencies, thus making them highly informative for genetic characterization and population structure analysis. The ISSR-based UPGMA dendrogram and principal coordinates analysis indicated that most cultivars exhibited genetic similarity, while a subset, including Jahesh, Fakhr, Sahel, and Hekmat, exhibited significant divergence, highlighting their potential as valuable resources for broadening the genetic base in breeding programs. Integration with a decision tree classifier enabled reproducible, rule-based cultivar identification, with specific loci such as Primer24_525 and Primer2_766 serving as diagnostic markers for cultivar differentiation.

These findings demonstrate that ISSR markers, in combination with machine learning methodologies, provide a cost-effective and practical framework for germplasm management and seed certification**.** Moreover, the study highlights that dominant markers, despite their limitations in distinguishing homozygous from heterozygous states, are advantageous for rapid genetic screening in resource-constrained breeding programs. Future research should prioritize the integration of ISSR data with co-dominant marker systems, such as SSRs and SNPs, as well as high-resolution genotyping platforms like GBS or NGS. This integration aims to enhance population structure analysis, improve authentication accuracy, and strengthen trait-marker associations. Furthermore, it could facilitate the creation of a comprehensive national cotton fingerprinting database, which would provide standardized DNA profiles for all registered cultivars, thereby supporting more efficient breeding, seed certification, and germplasm conservation efforts. Additionally, the genetically distinct cultivars identified in this study should be prioritized in breeding programs to enhance resilience, yield potential, and fiber quality.

## Author contributions

BP and RH conceived and design the study, performed the data analysis. ZG prepared the graphs and wrote the manuscript. The author(s) read and approved the final manuscript.

## Clinical trial number

Not applicable.

## Availability of data and materials

All relevant data are provided within the manuscript.

## Ethics approval and consent to participate

All experimental studies on plants were conducted in compliance with relevant institutional, national, and international guidelines and legislation.

## Consent for publication

Not applicable.

## Funding

This study was funded by 10.13039/501100007605Agricultural Biotechnology Research Institute of Iran (10.13039/501100007605ABRII) and Cotton Research Institute of Iran with number 013-07-0705-024-030597.

## Declaration of competing interest

The authors declare that there is not any conflict of interest.

## Data Availability

Data will be made available on request.
