# Supplementary material for: Integration of inter-simple sequence repeats with machine learning approach for diversity analysis and authentication of Iranian cotton cultivars
Source: Biochem Biophys Rep. 2026 Jan 6;45:102435. doi: 10.1016/j.bbrep.2025.102435 (PMC12808624; doi:10.1016/j.bbrep.2025.102435)
Supplement: Multimedia component 1 [file mmc1.docx]

**Supplementary Table S1.** ISSR primers used in this study

| Primer Code | Sequence (5′→3′) | Annealing Temperature (°C) | Expected Size Range (bp) | Reference |
| --- | --- | --- | --- | --- |
| ISSR-1 | GTGTGTGTGTGTTTGTT | 58 | 300–1500 | Zaki et al., 2023 |
| ISSR-2 | AGAGAGAGAGAGAGAGCC | 54 | 250–1200 | Zaki et al., 2023 |
| ISSR-3 | AGAGAGAGAGAGAAAGYA | 55 | 200–1400 | Zaki et al., 2023 |
| ISSR-4 | GGAGAGGAGAGGAGA | 50 | 350–1300 | Zaki et al., 2023 |
| ISSR-5 | GAGAGAGAGAGAGAGAYT | 52 | 200–1100 | Zaki et al., 2023 |
| ISSR-6 | ACACACACACACACACTG | 56 | 300–1250 | Dongre et al., 2007 |
| ISSR-7 | ACACACACACACACAAGT | 54 | 250–1400 | Adhikari et al., 2015 |
| ISSR-8 | AGTCGTAGTACACACACACACCC | 55 | 300–1350 | Adhikari et al., 2015 |
| ISSR-9 | CTCTCTCTCTCTCTCTCTG | 52 | 200–1000 | Adhikari et al., 2015 |
| ISSR-10 | AGAGAGAGAGAGAGAGYC | 53 | 300–1200 | Adhikari et al., 2015 |
| \ | (AC)₈YT | 54 | 200–1100 | Dongre et al., 2007 |
| ISSR-12 | (CT)₈RG | 56 | 250–1250 | Campbell et al., 2009 |
| ISSR-13 | (GA)₈YC | 52 | 250–1400 | Campbell et al., 2009 |
| ISSR-14 | (AG)₈C | 55 | 300–1500 | Campbell et al., 2009 |
